# Lattice Phase Field Model for Nanomaterials

**DOI:** 10.3390/ma14237317

**Published:** 2021-11-29

**Authors:** Pingping Wu, Yongfeng Liang

**Affiliations:** 1 Department of Materials Science and Engineering, Xiamen Institute of Technology, Xiamen 361021, China; 2 The Higher Educational Key Laboratory for Flexible Manufacturing Equipment Integration of Fujian Province, Xiamen Institute of Technology, Xiamen 361021, China; 3 State Key Laboratory for Advanced Metals and Materials, University of Science and Technology Beijing, Beijing 100083, China; liangyf@skl.ustb.edu.cn

**Keywords:** phase field model, ferroelectrics, ferromagnetics, grain growth

## Abstract

The lattice phase field model is developed to simulate microstructures of nanoscale materials. The grid spacing in simulation is rescaled and restricted to the lattice parameter of real materials. Two possible approaches are used to solve the phase field equations at the length scale of lattice parameter. Examples for lattice phase field modeling of complex nanostructures are presented to demonstrate the potential and capability of this model, including ferroelectric superlattice structure, ferromagnetic composites, and the grain growth process under stress. Advantages, disadvantages, and future directions with this phase field model are discussed briefly.

## 1. Introduction

Recent advances in phase field modeling have been effectively applied to quantitative evolutions of material microstructures, e.g., growth of dendrites and eutectics [1,2,3], solid state phase transformations [4,5], grain growth [4], nuclear materials [6], and ferromagnetic [7] and ferroelectric materials [8,9]. It has several advantages in the following aspects: (i) According to the physical properties studied and the chosen order of parameters, the phase field model can be used to describe a wide range of various microstructures. (ii) The phase field model can simultaneously perform the evolution of multi-order parameters in one simulation by calculating the coupling effects between different order parameters. (iii) The phase field model is generally considered a mesoscale simulation method; however, recent simulation works are approaching nanoscopic length scale and macroscopic length scale. (iv) It should be noted that the initial purpose of introducing phase field models is to avoid tracking the interfaces, which makes it easier to simulate the evolution of the microstructures. (v) The progress of recent works also shows that the phase field model can predict local structure evolution under scanning microscopy tips.

Over the last decades, new novel nanomaterials in various fields attracted much interest for their excellent physicochemical properties compared to bulk materials [10,11]. Some phase field simulation results for nanomaterials are summarized in previous review articles [7,8,9]. It should be noted that, as a phenomenological model for continuous media, the grid spacing for the phase field model can be very small, even smaller than the lattice parameter of real materials, e.g., [12,13]. We cannot say that the predicted simulation results of the microstructures are not correct because the statistical values of physical properties in simulations are reasonable. The major disadvantage comes from direct comparison with experimental results. Predicted order parameters on a scale smaller than the size of the lattice parameter cannot be observed in experiments, which leads to the continuous interfaces being unreasonable. The mismatch between the lattice parameter for nanomaterials and the grid spacing in the phase field model makes it more difficult to compare simulated results to the experimental works. From these points of view, we need to develop lattice phase field simulation techniques for nanomaterials. The standpoint of this article is that it is not appropriate to use the grid spacing under the Angstrom scale; the grid spacing should be rescaled and restricted to the lattice parameter for the nanomaterials.

To demonstrate how the model is constructed, it starts with the calculation of the interfacial energy in the phase field model and how to rescale the grid spacing to match the lattice parameter for real materials, followed by a detailed description of the simulation methods employed in this article. Two effective approaches are suggested to rescale the grid spacing in practice. Three specific complex cases of applying this model to nanostructured materials are presented, including ferroelectric superlattices, ferromagnetic composites, and the grain growth process under stress. The advantages and disadvantages of the lattice phase field model are summarized in this paper, and we also point out some potential applications for this model in the future. 

## 2. Phase Field Methods

**Figure 1:**
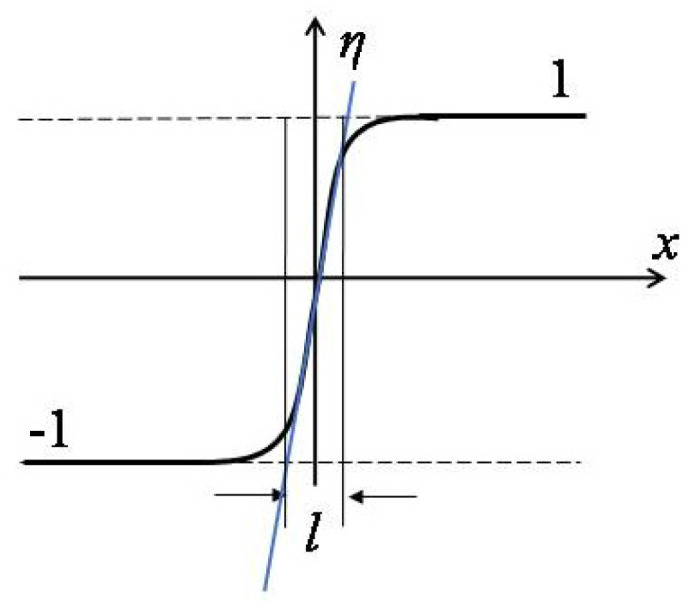
Schematic phase field profile across the interface.

The aim of the lattice phase field model is to connect the simulated grid spacing with the lattice parameter of real materials. First, let us see how to determine the real value of the grid size in the phase field model. One of the most key advantages of the phase field model is that this model avoids tracking phase boundaries during microstructure evolution, and interfaces can generate, disappear, or move according to changes in the total free energy of the material [4]. A schematic plot of the interface between two order parameters is shown in Figure 1. The average interfacial energy density, or average gradient energy density, for this simple model can be written as [4,14]

(1) $$\sigma =\frac{\kappa }{2}{\left(\nabla \eta \right)}^{2},$$

where κ is called the gradient energy coefficient, and η = +1/−1 is the order parameter describing the phase structure in simulation.

At equilibrium, the interface width *l*, illustrated in Figure 1, can be calculated by

(2) $$l=\sqrt{\frac{2\kappa }{\sigma }},$$

The interfacial energy density or the interface width can be determined from experimental measurements or first-principle calculations; thus the gradient energy coefficient κ can be deduced from (1) and (2). It should be pointed out that since the phase field model is a phenomenological model, the predicted domain size and its formed microstructure can be sensitive to the experimentally measured values or first-principle calculations.

Generally, the interface width should be larger than the grid spacing *dx* in simulation, i.e., *l* = N•*dx*, where N > 1. Otherwise, the interface becomes very sharp and leads to the grid-pinning phenomenon in simulation. Once the gradient energy coefficient and grid spacing are determined, the physical size of the material can be linked to the simulation size of the system.

Two approaches are suggested to rescale the grid spacing to the lattice parameter for real material in this paper. Firstly, a reference length *l*_0_ relevant to interfacial energy density was introduced, i.e., $l_{0}=\sqrt{2\kappa_{0}/\sigma_{0}}$, and the grid spacing *dx* = *n_x_*•*l*_0_ was introduced, where *n_x_* is a scale parameter. One simple way (Method 1) is to directly set the distance between grid points *dx*, *dy,* and *dz* to the lattice parameter for the simulated materials while *l*_0_ stays the same. In other words, the scale parameter changes. The second approach (Method 2) recognizes that the grid spacing also can be adjusted by the gradient energy coefficient; for example, reducing the interface energy coefficient κ_0_ results in a decrease in *l*_0_, and, therefore, the same interface requires more grids in simulation, with reduced grid spacing *dx*. The main disadvantage of the two methods is that the simulation is quite memory- and CPU time-consuming for a system with many grids, and the computer memory is always limited.

Combing the interfacial energy and other chemical bulk energies gives phase field dynamic equations for conserved or non-conserved systems, recognized as Cahn–Hilliard equations or Ginzburg–Landau (Allen–Cahn) equations [15], respectively:

(3) $$\frac{\partial \eta }{\partial t}=\nabla \cdot \left(M\nabla \frac{\delta E_{tot}}{\delta \eta }\right),$$

(4) $$\frac{\partial \eta }{\partial t}=-L\frac{\delta E_{tot}}{\delta \eta },$$

where *L* and *M* are the dynamic coefficients for conserved and non-conserved equations, respectively. *E_tot_* represents the total free energy of the system. The phase field equations can be solved by several numerical algorithms, e.g., the finite difference method, the semi-implicit Fourier space method, the finite element method, etc. [15].

For a ferroelectric system, the evolution of the ferroelectric polarization distribution is governed by the Ginzburg–Landau equation, [8,16].

(5) $$\frac{\partial P}{\partial t}=-L\frac{\delta E}{\delta P},$$

where P_i_ (i = 1, 2, 3) is the spontaneous polarization, *L* is the kinetic parameter, and E is the total free energy of the ferroelectric system, which includes the contributions of Landau free energy, interfacial energy, electric energy, and elastic energy, i.e.,

(6) $$E=\int^{\;}\left[\begin{array}{c} \frac{1}{2}a_{ij}P_{i}P_{j}+\frac{1}{4}a_{ijkl}P_{i}P_{j}P_{k}P_{l}+\ldots +\frac{1}{2}G_{ijkl}\frac{\partial P_{i}}{\partial x_{j}}\frac{\partial P_{k}}{\partial x_{l}}-\frac{1}{2}\varepsilon_{b}\varepsilon_{0}E_{i}^{2}\\ -E_{i}P_{i}+\frac{1}{2}C_{ijkl}\left(\varepsilon_{ij}-Q_{ijkl}P_{k}P_{l}\right)\left(\varepsilon_{kl}-Q_{klij}P_{i}P_{j}\right) \end{array}\right]\mathrm{dV},$$

where a*_ij_*, a*_ijkl_* are Landau expansion coefficients, *G_ijkl_* is the gradient energy coefficient, and ε_0_ and ε_b_ are the vacuum permittivity and the background relative dielectric permittivity. *E_i_* is the external electric field. *C_ijkl_* is the elastic stiffness tensor. *ε_ij_* is the total strain and *Q_ijkl_* is the electrostrictive coefficient. With all the energetic contributions, the evolution of the ferroelectric domain structure can be obtained by solving Equation (5) using the Fourier spectral method [17] and Khachaturyan’s elastic theory [18].

For a ferromagnetic system, the ferromagnetic domain structure evolution is governed by the Landau–Lifshitz–Gilbert equation, [7,19].

(7) $$\left(1+\alpha^{2}\right)\frac{\partial M}{\partial t}=-\gamma_{0}M\times H_{\mathrm{eff}}-\frac{\gamma_{0^{\alpha }}}{M_{s}}M\times \left(M\times H_{\mathrm{eff}}\right),$$

where M_i_ (i = 1, 2, 3) is the spontaneous magnetization, α is the damping factor, t is time, γ_0_ is called gyromagnetic ratio, M_s_ is saturation magnetization, and the effective magnetic field H_eff_ = −(1/μ_0_)·∂E/∂M, where E is the total free energy of a magnetic system, which includes the contributions of anisotropy energy, exchange energy, magnetostatic energy, and external field energy, i.e.,

(8) $$E=\int^{\;}\left[\begin{array}{c} K_{1}\left(m_{1}^{2}m_{2}^{2}+m_{1}^{2}m_{3}^{2}+m_{2}^{2}m_{3}^{2}\right)+K_{2}m_{1}^{2}m_{2}^{2}m_{3}^{2}\\ +A{\left(\nabla m\right)}^{2}-\frac{1}{2}\mu_{0}M_{s}H_{d}\cdot m-\mu_{0}M_{s}H_{ex}\cdot m \end{array}\right]\mathrm{dV},$$

where K_1_, K_2_ are anisotropy constants, m_i_ = M_i_/M_s_, A is called the exchange coupling constant, μ_0_ is the vacuum magnetic permeability, H_d_ and H_ex_ are the demagnetization field and external magnetic field, and V is the representation volume. With all the energetic contributions, the evolution of the ferromagnetic domain structure can be obtained by solving Equation (7) using the Gauss–Seidel projection method [20].

For a multi-grained system, the evolution of the grain structure can be described by a set of Ginzburg–Landau equations,

(9) $$\frac{\partial \eta_{i}}{\partial t}=-L\frac{\delta E}{\delta \eta_{i}},$$

where the η_i_ (i = 1, 2, 3…*p*) are called the orientation field variables representing the orientation of grains. In this work, a random set of orientations of grains between [−45°, 45°] is connected to the phase field order parameter η_i_. *L* is the kinetic parameter for grain migration, and E is the total free energy of the multi-grain system, which includes the contributions of the chemical bulk free energy, interfacial energy, and elastic energy, i.e.,

(10) $$E=\int^{\;}\left[\begin{array}{c} \sum_{i=1}^{p}\left(\frac{\alpha \left(T-T_{c}\right)}{2}\eta_{i}^{2}+\frac{\beta }{4}\eta_{i}^{4}\right)+\gamma \sum_{i=1}^{p}\sum_{j\neq i}^{p}\eta_{i}\eta_{j}\\ +\sum_{i=1}^{p}\frac{\kappa_{i}}{2}{\left(\nabla \eta_{i}\left(r\right)\right)}^{2}+\frac{1}{2}C_{ijkl}\left(\varepsilon_{ij}-\varepsilon_{ij}^{0}\right)\left(\varepsilon_{kl}-\varepsilon_{kl}^{0}\right) \end{array}\right]\mathrm{dV},$$

where α, β, and γ are Landau bulk free energy coefficients, T and T_c_ represent the temperature and phase transition temperature, κ_i_ is the gradient energy coefficient, C_ijkl_ is the elastic constant, and ε_ij_ and ε_ij_^0^ represent the microelastic total strain and local eigen-strain in crystal. In this paper, the periodic boundary conditions are employed in all the simulations.

## 3. Simulation Results

In this section, the lattice phase field model is employed to study various materials systems. These simulation works are programmed and performed on the platform of the GNU Octave software package. The phase field equations are solved using the semi-implicit Fourier space method, and all the simulated microstructures at late stages of the evolution process are close to the equilibrium state.

**Figure 2:**
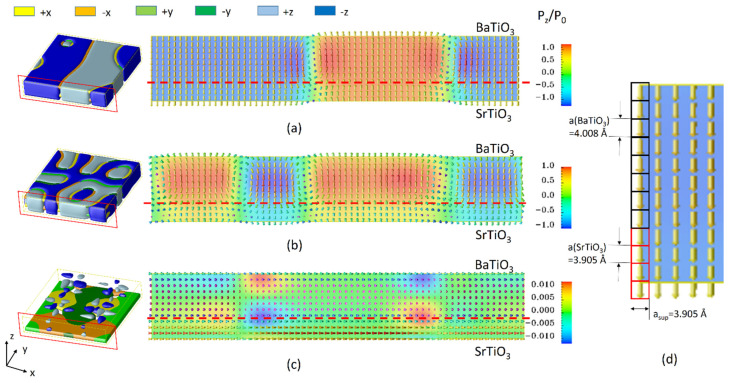
The simulated superlattice ferroelectric domain structures (left) and the vector plot of polarization in the cross-section plane (right): (**a**) under the fully commensurate condition, (**b**) under a partially relaxed condition, and (**c**) under the fully relaxed condition. (**d**) A detailed vector plot of (**a**) with lattice grids. Please note the grid spacing Δ*x*_1_ = Δ*x*_2_ = a_sup_, Δ*x*_3_ = 3.905 Å forthe SrTiO_3_ layer, andΔ*x*_3_ = 4.008 Å for the BaTiO_3_ layer.

Firstly, consider a ferroelectric superlattice structure of (SrTiO_3_)_4_/(BaTiO_3_)_8_. This oxide nano-heterostructure periodically grown at the atomic level has attracted great attention due to its adjustable dielectric and ferroelectric properties and its potential applications for electronic memory devices. It should be noted that the ferroelectric properties of the superlattice structure can be highly influenced by the relaxation condition between the thin film–substrate interface. For a heterostructure thin film grown on a certain substrate, lattice parameters of the film and substrate materials can be different. For a ferroelectric thin film constrained by a substrate, the thin film is considered in a commensurate condition. If the lattice mismatch between the thin film and substrate is larger, this results in a relaxation of the film material, which accommodates interfacial strain in the system and leads to a partially relaxed or fully relaxed film. Figure 2a–c show the domain structures and polarization vector plots of a single period of (SrTiO_3_)_4_/(BaTiO_3_)_8_ grown on a SrTiO_3_ substrate at fully commensurate, partially relaxed, and fully relaxed interfacial coherency conditions, respectively. The ferroelectric domain structures are performed after 12,000 time steps of evolution. In the simulation, the simulation cell is discretized by 64Δ*x*_1_ × 64Δ*x*_2_ × 12Δ*x*_3_ grids, where each grid represents a single lattice, and Method 1 is employed in this case. At room temperature, the in-plane lattice parameters of the superlattice a_sup_ are 3.905, 3.946, and 3.969 Å for fully commensurate, partially relaxed, and fully relaxed relaxation conditions [21].Thus the in-plane grid spacing is set as Δ*x*_1_ = Δ*x*_2_ = a_sup_, for the SrTiO_3_ layer Δ*x*_3_ = a(SrTiO_3_) = 3.905 Å, and for BaTiO_3_ layer Δ*x*_3_ =a(SrTiO_3_) = 4.008 Å. The material parameters for BaTiO_3_ and SrTiO_3_ are described in Ref. [21].The reduced gradient coefficient g_11_ = G_11_/G_110_ is chosen to be 2.0, g_12_ = 0, g_44_ = g’_44_ = 1.0, G_110_ = a_0_*l*_0_^2^, *l*_0_ = 1×10^−9^ m, a_0_ = 0.371 × 10^8^ C^−2^m^2^N, and P_0_ = 0.26 C/m^2^ at room temperature. For 180-degree domain walls, the domain wall energy density of BaTiO_3_ is evaluated to be about 2g_11_a_0_*l*_0_P_0_^2^ = 0.01 Nm^−1^, which is consistent with existing experimental measurement 10 erg/cm^2^ reported by Merz [22]. Since the time length of the relaxation process is significantly larger than that of the dynamical process in phase field simulation, the initial grid spacing is unchanged in the calculation process. The predicted domain structures agree remarkably well with the experimental observation by piezoelectric force microscopy [23].

In the vector plot, each vector represents a single polarization generated withinthe crystal lattice. The grid spacing between each neighboring vector is exactly the lattice parameter for real materials. It is worth noting that the length in the *x*-direction of the vector plot under the three types of constraints is different due to the different grid spacing that is employed, and the length in the *y*-direction of the vector plot is subject to the lattice parameter of the ferroelectric materials, as shown in Figure 2d. Suffering from the in-plane strain generated by the interfacial coherency, the polarization rotates from the out-of-plane direction to the in-plane direction and orthorhombic phases are formed in ferroelectric domain structures. Thus this model can simulate the structure of polarization distributions at the atomistic scale and monitor the polarization development in the crystal lattice of superlattice ferroelectrics. Compared to our previous work of [21], this model predicted local polarization in the crystalline lattice of the ferroelectric body. These simulated structures can better compare with experimental measurements at the atomic level, e.g., domain structure, domain wall, local polarization distribution, etc.

**Figure 3:**
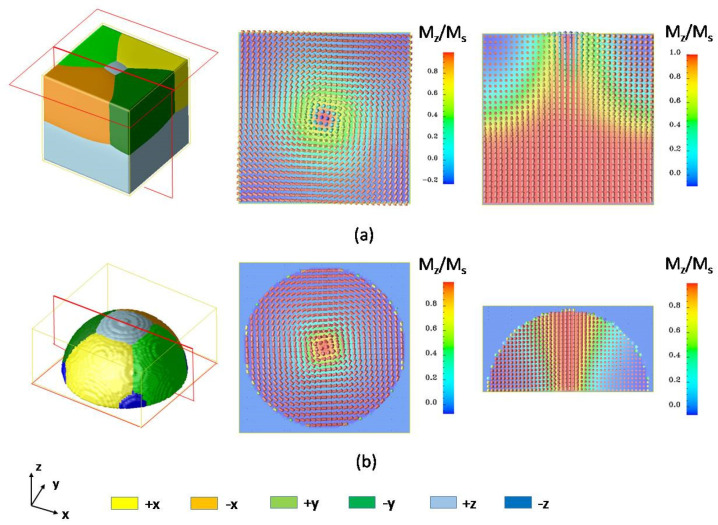
The simulated ferromagnetic domain structure and magnetization distributions of composite FePt/Fe with (**a**) bilayer structure (**b**) core–shell structure.

Next, consider a ferromagnetic composite of FePt/Fe. Figure 3a illustrates ferromagnetic domain structures after 8000 computational time steps of evolution. The initial domain structure is magnetized to saturation along the z-direction, and then we place an external field of 500 kA/m along the −z-direction. The two-layered magnetic nanodot structure consists of an iron soft magnetic layer and an FePt hard magnetic layer. This structure is called an exchange coupled composite structure and is widely used in ultra-high-density magnetic memory devices [19]. During the switching process, the soft magnetic layer can help reverse the magnetization of the whole structure while the hard layer can keep the structure stable at a zero field. This composite structure can greatly reduce the switching field, decrease the energy required in the writing process, and increase the density of the magnetic memory bit. The simulation is performed on a 64 × 64 × 64 mesh with the grid size of the FePt hard layer and Fe soft layer set to 0.386 nm [24] and 0.287 nm [25], respectively. The material parameters for FePt and iron can be found in Ref. [19]. Method 2 is employed to change the interfacial coefficient to adjust the grid size *ld* in the model to match the lattice parameters of an *L*1_0_ phase FePt alloy and bcc iron. In magnetic simulations, the exchange energy, similar to the interfacial energy, plays a role in controlling the thickness of the magnetic domain wall. A reduced exchange stiffness constant A* was introduced to connect the grid size *ld* and the exchange stiffness constant between two spins, and A* is defined as A* = 2A/(μ_0_M_s_^2^*ld*^2^), where A is the exchange stiffness constant. For Fe, A = 2 × 10^−11^, and for FePt, A = 1 × 10^−11^; thus the domain wall width, or the exchange length of the hard layer, is around *l*_exch_ = 2.43 nm, which is consistent with experimental results by Huang et al. [26]. Using Method 2, since we set the grid size *ld*(Fe) = 0.287 nm and *ld*(FePt) = 0.386 nm, the reduced exchange stiffness constants are chosen to be A*(Fe) = 155.78 and A*(FePt) = 96.887 in the simulation. A similar calculation process also can be found in Ref. [27].

Another advantage of exchange coupled composites is that the structure is easy to be designed for better performance. The simulated structure of the composite can be very flexible. Figure 3b shows another popular structure used for bit-patterned media: the core–shell structure, with an FePt nanodot core covered by an iron layer as shell, which was found to be very efficient in reducing the switching field. The nominal thicknesses of the FePt core and the Fe shell are8 nm and 5 nm, respectively.

As the magnetization is constrained to lie in the thin film plane by magnetocrystalline anisotropy energy, vortex ferromagnetic domain configurations are found in the soft magnetic layer for both double-layered and core–shelled structures. The predicted vortex structure is experimentally observed in a similar double-layered *L*1_0_−FePt/permalloy structure by Zhou et al. [28]. The vector plots on the right of Figure 3 show the magnetization distributions in the *xy* plane and the *xz* plane during the switching process. In the following process, the magnetization in the FePt layer starts rotating due to the exchange coupling effect between the soft layer and hard layer. This effect requires the magnetizations to tend to align in the same direction, and finally, the magnetization of the whole structure will be reversed. In this case, the distance between the two neighboring vectors is different since the gradient coefficient changes. This model can also integrate with elastic energy considering the magnetostrictions or magnetic shape memory devices. The gradient energy coefficients can reflect lattice shape and change to accurately predict the magnetic or mechanical properties of nanomaterials.

For the last case, consider a nano-scaled grain growth process under tensile stress. The nano-grained metals have attracted great attention due to their excellent properties for the structure designed [29,30]. Since the grain growth process is widely simulated through the phase field model [4,31,32], this lattice model can take into account the grain lattice direction in the elastic contribution of the total free energy. It should be noted that no specific material is employed. The materials coefficients for phase field modeling are the same as that in Ref. [31]: α = β = γ = 1.0, *L* = 1.0 for all the grains, and the cubic phase crystal with elastic coefficients C_11_ = 450 × 10^9^ N/m^2^, C_12_= 150 × 10^9^ N/m^2^, and C_44_ = 300 × 10^9^ N/m^2^. A 2D model with system size of 64 × 64 mesh grids is employed in this case, and the gradient coefficient is set to be 1.0. The grid spacing is chosen to be *dx* = *dy* = 1.0. Both Method 1 and Method 2 can be applied in this example. If using Method 1, one can directly set *dx* equal to the lattice parameter of the aim material. If Method 2 is used with fixed grid spacing, the gradient energy coefficient κ can be determined by Equation (2).

**Figure 4:**
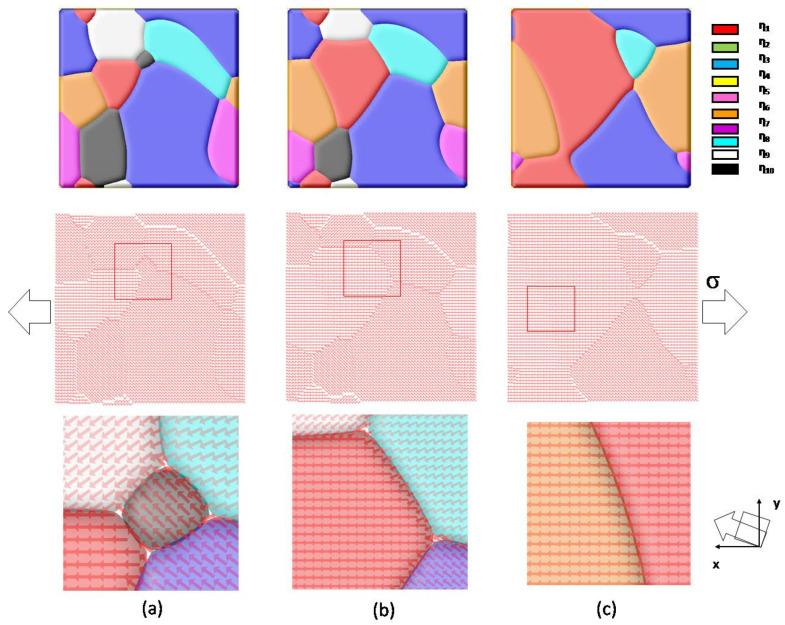
(**a**–**c**) The grain growth process at a tensile stress of 10 MPa along the *x*-direction. The vectors indicate the orientation of grains, which is assumed to be −45°~45°with a random distribution. In this work, the orientation of grains η_1_~η_10_ are 0°, −7.6°, 35.9°, 9.7°, −17.1°, 2.88°, 23.1°, −23.7°, −35.8°, and 43.7°, respectively.

Figure 4a–c illustrate the evolution of multi-grain material at a tensile stress of 10 MPa after 5000 steps. The order parameters η_1_~η_10_ represent grains with a given crystallographic orientation. In this 2D simulation, a vector of constant length denotes the angle between the grain direction and the *x*-axis. Under tensile stress along the *x*-direction, one can see that grain boundary migration occurs, the evolution of simulated grain structure reveals that the energetically favorable grain starts growing, and unfavorable orientated grain disappears. It is also interesting to note that a small-angle-grain-boundary is observed between the red-colored grain (with a misorientation angle of 0°), and orange-colored grain (with a misorientation angle of 2.88°), as shown in Figure 4c. This grain boundary almost keeps static, since the elastic energy contributions of neighboring grains are similar under applied stress. This multi-grain model also can be integrated with the previous ferroelectric/ferromagnetic models to study the physical properties of nanoscale multi-grained materials.

## 4. Summary Remarks

In this paper, a lattice phase field model is developed to investigate microstructures of materials at the nanoscale, where the grid spacing is carefully controlled to match the lattice parameters of the crystalline material. Two methods are suggested in this work to adjust the gradient spacing; one is to directly control the grid spacing *dx*, *dy*, and *dz*, and the other is adjusting the gradient energy coefficients. These simulation examples allow convenient application of this lattice phase field model to various microstructure evolution process at the nanoscale, such as ferroelectric, ferromagnetic, and nano-grained materials.

The main advantage of the lattice phase field model is that it avoids unphysical predicted values of the order parameter in the case where the grid spacing is set smaller than the atomic spacing. Using this criterion, the grid spacing would correspond to the lattice parameter of materials in real space. Thus the predicted values or images can be directly compared to the experimental results at the atomic scale. The most obvious disadvantage for this model is the continuum phenomenological nature of the phase field method. The input coefficients are very sensitive to the experimental measurements or predicted values from the first-principle calculation. It also should be pointed out that 3D structures require more computing power than traditional phase field simulation. A future direction is to employ this lattice phase field model to study or design nanostructured materials or nanoscale devices in various application areas.
